# Preliminary Observations of Bilateral Neovascular Age-Related Macular Degeneration Progression: A Real-World Retrospective Case Series

**DOI:** 10.3390/jcm15114051

**Published:** 2026-05-24

**Authors:** Ching-Han Tseng, Meng-Yin Lin, Du-I Chiou, Chi-Hsin Hsu, Chia-Min Wu

**Affiliations:** 1 Department of Ophthalmology, Shuang Ho Hospital, Taipei Medical University, No. 291, Zhongzheng Rd., Zhonghe District, New Taipei City 23561, Taiwan; 25186@s.tmu.edu.tw (C.-H.T.); 11069@s.tmu.edu.tw (C.-H.H.); 2 Department of Ophthalmology, Taipei Municipal Wan Fang Hospital, Taipei Medical University, Taipei 116035, Taiwan

**Keywords:** bilateral neovascular age related macular degeneration, second-eye conversion, treatment burden, patient monitoring, retinal pigment epithelium abnormalities

## Abstract

**Background**: This study investigated the clinical timeline, patient monitoring behaviors, and cumulative bilateral treatment burden in patients with bilateral neovascular age-related macular degeneration. **Methods**: We retrospectively analyzed follow-up patterns and treatment intensity from first-eye (FE) diagnosis to second-eye (SE) conversion. **Results**: SE conversion occurred within a mean of 2.0 years in the FE-active group (62.5%) while the FE remained exudative, contrasting with 6.2 years in the FE-inactive group (37.5%). Upon SE conversion, the total annual intravitreal injection burden escalated 3.4-fold (*p* = 0.002). Notably, the FE-inactive group exhibited numerically lower annual outpatient visit counts (4.40 ± 2.71 vs. 10.29 ± 5.02; *p* = 0.116), which potentially widened the monitoring window. Additionally, baseline SE retinal pigment epithelium (RPE) abnormalities independently predicted progression (aOR: 19.04; *p* = 0.032). **Conclusions**: While previous literature focuses on individual eyes, our findings highlight a vigilance gap in SE detection based on FE status. Clinicians must maintain proactive surveillance for patients with baseline SE RPE abnormalities, particularly when FE stability or next-generation long-acting therapies extend clinic intervals. Due to the limited sample size, these preliminary findings warrant validation in larger prospective cohorts.

## 1. Introduction

Age-related macular degeneration (AMD) is a leading cause of severe visual loss and blindness in aging people worldwide [1,2,3]. The prevalence of late AMD, defined by the presence of neovascular AMD (nAMD) or geographic atrophy, ranges from approximately 1.7% to 1.9% and increases markedly with age [4,5]. Among these conditions, nAMD accounts for only 10–15% of all AMD cases but is responsible for the majority of AMD-related severe vision loss as a result of choroidal neovascularization [6,7]. nAMD is currently the subtype with the most effective therapeutic options. Intravitreal injections (IVI) of anti-vascular endothelial growth factor agents (anti-VEGF) constitute the standard of care for nAMD and have been demonstrated to preserve or maintain visual acuity in the majority of treated patients [8,9]. However, long-term treatment may be associated with substantial direct medical costs and indirect psychosocial burdens [10,11,12,13]. In addition, although progression from unilateral to bilateral nAMD affects a minority of patients, it may be associated with increased treatment burden, greater visual impairment, and elevated healthcare utilization and societal costs; however, real-world quantitative data characterizing this progression remain limited [14,15,16,17,18]. Taiwan’s National Health Insurance (NHI) was launched in 1995 and has achieved 99.9% universal health coverage, providing equitable access to healthcare for all citizens and legal residents [19]. The NHI program in Taiwan provides a comprehensive reimbursement framework for intravitreal anti-VEGF therapy in nAMD. Ranibizumab was initially reimbursed for nAMD in 2011, with later policy revisions expanding eligible agents and revising injection limits and treatment duration [7,20,21,22]. Furthermore, the NHI expanded its reimbursement policy in 2020 to allow up to 14 injections per eye [22]. In Taiwan, the comprehensive coverage provided by the NHI system ensures that the majority of patients with nAMD or polypoidal choroidal vasculopathy (PCV) can receive anti-VEGF therapy according to established clinical guidelines. This protocol typically consists of a loading phase of three monthly injections followed by a pro re nata (PRN) regimen, which remains accessible to patients regardless of whether they have unilateral or bilateral involvement [7,23]. Since the accessibility of treatment is not constrained by individual economic status under this universal healthcare framework, the direct economic impact of therapy was not specifically examined in this study.

This study incorporates several established risk factors for second-eye (SE) involvement within an Asian context, including advanced age, retinal pigment epithelium (RPE) abnormalities, tobacco use, and systemic comorbidities such as hypertension. These variables are recognized in the literature as significant predictors for progression to nAMD [16,24,25].

The clinical landscape of nAMD in Asia is unique, characterized by a high prevalence of PCV [26,27]. Although some literature suggests that PCV is associated with a lower bilateral conversion rate compared to typical nAMD, analyzing these phenotypes collectively reflects the real-world complexity faced by Asian practitioners [28].

While previous studies have noted that the SE often presents with better visual acuity at conversion and receives fewer injections than the first eye (FE), the cumulative bilateral treatment burden and the FE status (active vs. inactive) during SE conversion remain less explored [18,29]. Consequently, this study seeks to evaluate bilateral conversion within an Asian cohort capturing diverse clinical phenotypes, including PCV. Beyond these objectives, we examine the potential longitudinal shift in annual cumulative injection burden and the logistical challenges of synchronized therapy from a granular perspective. By evaluating these evolving treatment demands, our findings may provide preliminary insights to inform more sustainable monitoring and treatment frameworks for the SE.

## 2. Materials and Methods

### 2.1. Study Design and Patient Selection

This retrospective case series was conducted by a single retinal specialist (C.M.W.) at Taipei Medical University Shuang Ho Hospital. Consecutive patients diagnosed with unilateral nAMD, including the PCV subtype, and treated with intravitreal anti-VEGF injections between January 2016 and December 2024 were enrolled. Inclusion required: (1) age ≥ 50 years; (2) active macular neovascularization (MNV) confirmed by optical coherence tomography (OCT) and fluorescein angiography (FA), with or without indocyanine green angiography (ICGA); and (3) a minimum follow-up of 6 months. Cases with bilateral involvement at baseline or other confounding maculopathies were excluded.

The diagnosis and subtype classification of nAMD were established using multimodal imaging(MMI), including color fundus photography, FA, ICGA, and OCT, adhering to the consensus established by Taiwan retinal specialists [7]. Specifically, active nAMD was defined by the presence of exudative signs, including intraretinal fluid (IRF), subretinal fluid (SRF), or active leakage on FA, as per the Consensus on Neovascular AMD guidelines and the established local consensus among Taiwan retinal specialists [7,30]. In contrast, inactive nAMD or submacular fibrosis was characterized by the absence of fluid and the presence of a well-demarcated, yellowish-white macular lesion on fundus photography. On OCT, this corresponded to persistent, dense, subretinal hyperreflective material (SHRM) with associated retinal pigment epithelium (RPE) disruption and overlying retinal atrophy, representing the cicatricial end-stage of the disease [30,31]. This distinction categorized eyes with active neovascularization as exudative, while those with established submacular fibrosis were classified as having irreversible structural damage.

In this study, nine patients were diagnosed with PCV (seven with unilateral and two with bilateral nAMD). The diagnosis in all nine cases was confirmed by identifying focal subretinal polyps or branching vascular networks on ICGA, which were then cross-validated with validated OCT criteria including sharp-peaked pigment epithelial detachment (PED) or the ‘double-layer sign’ in accordance with the Everest criteria [32]. Furthermore, RPE abnormalities in the SE were collectively defined as the presence of RPE detachment or RPE defects, including incomplete or complete RPE and outer retinal atrophy. These alterations were identified using MMI, with classification based on the Age-Related Eye Disease Study Report Number 6 for pigmentary changes, the Classification of Atrophy Meetings consensus for RPE atrophy, and the Consensus on Neovascular Age-Related Macular Degeneration Nomenclature for RPE detachment subtypes (serous or drusenoid) as observed on structural OCT [30,33].

To ensure diagnostic consistency, all imaging data were independently graded by two retinal specialists (C.H.T. and C.M.W.). Discrepancies in subtyping or progression status were resolved by consensus.

Most treatments followed Taiwan NHI reimbursement criteria, which require angiographic confirmation (FA with or without ICGA), OCT evidence of disease activity, and BCVA between 20/400 and 20/40, permitting up to 14 reimbursed injections per eye over a five-year period [22] Ranibizumab (0.5 mg) and aflibercept (2 mg) were the primary agents used. Notably, following its NHI approval in January 2024, faricimab (6 mg) was administered to the SE of one patient in the bilateral cohort for four doses, while aflibercept remained the primary agent for the FE. Additionally, bevacizumab (1.25 mg) was used in a limited number of self-paid injections.

Anti-VEGF therapy was generally initiated with three monthly loading injections, followed by 1–2-month follow-up and PRN retreatment based on changes in BCVA and central foveal thickness (CFT) [7,34]. The PRN regimen was guided by monthly BCVA and OCT assessments. Retreatment was triggered by: (1) BCVA loss ≥ 5 letters; (2) new or persistent SRF/IRF or PED; (3) CFT increase ≥ 100 μm; or (4) new macular hemorrhage. Treatment was deferred if visual loss was <5 letters and anatomical findings remained stable (CFT increase < 100 μm, no new hemorrhage, and fluid stability over two consecutive visits).

In patients with bilateral active nAMD, PRN was applied when disease activity was detected in either eye, with same-day bilateral injections considered when appropriate after obtaining patient consent.

All injections were performed under topical proparacaine anesthesia following 5% povidone–iodine disinfection. Post injection, topical tobramycin/dexamethasone drops were prescribed four times daily for three days. For same-day bilateral injections, each eye was prepared and treated separately using independent sterile instruments. Medical records were reviewed to collect data on systemic comorbidities, IVI history, and ocular parameters for analysis. The study protocol was approved by the Institutional Review Board of Taipei Medical University (TMU-JIRB No. N202308054) and was conducted in accordance with the Declaration of Helsinki.

### 2.2. Data Collection

Initially, 39 patients with unilateral nAMD were identified for potential inclusion. A total of 13 patients were subsequently excluded based on the following criteria: 10 patients presented with concomitant ocular pathologies or medical histories that could confound the results, including chronic central serous chorioretinopathy (*n* = 2), severe diabetic retinopathy (*n* = 2), retinal vein occlusion (*n* = 3), and severe hypertensive retinopathy (*n* = 1), while two cases were excluded due to delayed presentation. In addition, 1 patient was excluded due to an unclassified nAMD subtype and inconclusive imaging for retinal angiomatous proliferation without ICGA confirmation. Finally, 2 patients were excluded due to loss to follow-up. Consequently, a final cohort of 26 patients was included in the analysis.

All data were independently retrieved, reviewed, and verified by two reviewers (C.H.T. and C.M.W.). Collected variables included sex, age, laterality, systemic comorbidities (diabetes mellitus, hypertension, and dyslipidemia), tobacco history (current or former smoker vs. never smoker), IVI frequency, anti-VEGFs used (bevacizumab 1.25 mg, ranibizumab 0.5 mg, aflibercept 2 mg, and faricimab 6 mg), and follow-up duration. Follow-up duration was defined as the interval from first-eye (FE) nAMD diagnosis to the last clinic visit in the unilateral group, and to SE nAMD diagnosis in the bilateral group.

Ocular parameters measured included BCVA assessed with a tumbling E chart, intraocular pressure (IOP) measured using a noncontact tonometer (Nidek NT-530P Tonopachy, NIDEK Co., Ltd., Gamagori, Japan), and macular morphology, central foveal thickness (CFT) and baseline structural abnormalities of the RPE with or without drusen in the SE evaluated using spectral-domain OCT (Optovue Avanti widefield OCT, Ophthalmicmart, Singapore).

### 2.3. Statistical Analysis

Patients were classified into two groups, unilateral nAMD group and bilateral nAMD group based on their status at the last follow-up. Baseline characteristics between the two groups were compared using the independent samples *t*-test for continuous variables and Fisher’s exact test or the chi-square test, as appropriate, depending on cell counts for categorical variables. BCVA assessed with the tumbling E chart was approximated to the logarithm of the minimum angle of resolution (logMAR) values for descriptive and comparative analyses.

Univariate and multivariable binary logistic regression analyses were performed to evaluate the association between each potential risk factor and the development of bilateral nAMD.

All statistical analyses were performed using IBM SPSS Statistics, version 27 (IBM Corp., Armonk, NY, USA). A *p*-value of <0.05 was considered statistically significant.

## 3. Results

In Table 1, a total of 26 patients were included in the study, comprising 18 patients in the unilateral nAMD group and 8 patients in the bilateral nAMD group. The mean age was 72.69 ± 9.39 years in the unilateral group and 70.12 ± 5.99 years in the bilateral group (*p* = 0.49). The proportion of male patients was 50% in the unilateral group and 87.5% in the bilateral group (*p* = 0.09). The mean follow-up duration was approximately 2.91 ± 2.23 years and 3.65 ± 3.16 years, respectively (*p* = 0.51).

In the unilateral nAMD group, 11 out of 18 patients (61.1%) were diagnosed with typical nAMD and 7 with PCV (38.9%). In the bilateral nAMD group, 6 patients were diagnosed with typical nAMD (75%) and 2 with PCV (25%). Two patients (11.1%) in the unilateral group and four (50.0%) in the bilateral group had a history of tobacco use (*p* = 0.07). Additionally, seven patients in both the unilateral (38.9%) and bilateral (87.5%) groups exhibited baseline subfoveal RPE changes in the SE (*p* = 0.02).

Univariate logistic regression analysis of the full cohort identified a history of tobacco use as significantly associated with progression from unilateral to bilateral nAMD (cOR = 8.00, 95% CI: 1.061–60.324, *p* = 0.04). Similarly, the presence of a baseline SE RPE abnormality was significantly associated with bilateral involvement (cOR = 11.00, 95% CI: 1.103–109.674, *p* = 0.04). Male sex showed a trend toward association (*p* = 0.06), whereas age, diabetes mellitus, hypertension, and dyslipidemia were not significantly associated (Table 2). In the primary multivariable model (adjusted for sex and tobacco use), baseline SE RPE abnormalities remained the only significant independent predictor for bilateral progression (aOR 19.04, 95% CI 1.287–281.535, *p* = 0.032) (Table 2). To evaluate the robustness of these findings, a sensitivity analysis was performed by excluding patients diagnosed with PCV. In this subgroup univariate analysis, associations with sex (*p* = 0.044), baseline SE RPE abnormalities (*p* = 0.060), and tobacco use (*p* = 0.083) remained close to or reached statistical significance, consistent with the primary findings. However, these variables did not retain significance in the subsequent multibariable model for the non-PCV subgroup. This lack of multivariable significance is likely attributable to the further reduction in sample size and limited statistical power, which is evidenced by high standard errors and model instability, rather than a lack of biological association.

Table 3 summarizes clinical characteristics of eight patients who progressed to bilateral nAMD and compares their IVI frequency before and after the SE diagnosis. The interval between FE and SE involvement varied substantially, ranging from 0.7 to 8 years, mean 3.6 years. At SE diagnosis, the FE remained exudative in five patients (62.5%) with a mean conversion time of 2.0 years, while the FE was inactive in three patients (37.5%) with a mean conversion time of 6.2 years. Although not reaching statistical significance (*p* = 0.116), the active FE group showed a higher numerical frequency of outpatient visits between FE diagnosis and SE conversion compared to the inactive FE group (10.29 ± 5.02 vs. 4.40 ± 2.71). Notably, 60% (3/5) of patients in the active FE group sought medical attention earlier than scheduled due to self-perceived visual changes in the SE.

Further analysis revealed that the annual cumulative IVI frequency significantly increased from 2.25 ± 2.55 injections (mean ± SD) during the unilateral phase to 7.75 ± 3.20 injections (mean ± SD) after second-eye involvement (*p* = 0.002). This represents an average 3.4-fold increase in the annual cumulative treatment burden for this subgroup. These treatment regimens primarily utilized ranibizumab and aflibercept 2 mg. Notably, upon the diagnosis of bilateral activity, five patients (62.5%) required bilateral injections to manage concurrent exudative activity in both eyes.

Overall, the development of bilateral nAMD was associated with increased cumulative treatment burden, as reflected by more frequent injections and the need for simultaneous bilateral therapy in the majority of patients.

## 4. Discussion

This preliminary retrospective study provides clinically relevant insights into the progression from unilateral to bilateral nAMD in a real-world Taiwanese cohort. We observed a SE conversion rate of 31% (8/26 patients) over a mean follow-up of 3.6 years. While this incidence is comparable to Western data—where cumulative incidences range from 32% to 40%—it remains notably higher than the 9.4% to 18.4% reported in various Asian epidemiological studies [16,18,28,35,36]. Nevertheless, in our cohort, PCV was more prevalent in the unilateral group than in the bilateral group (38.9% vs. 25.0%). Although not statistically significant, this trend aligns with established evidence that PCV carries a lower propensity for bilateral conversion compared to typical nAMD, particularly in Asian populations [28,37]. Such phenotypic heterogeneity may explain why established risk factors, such as advanced age and poor baseline visual acuity, did not emerge as significant predictors in our study. In this mixed Asian cohort, the initial clinical status of the FE appears to be a less reliable indicator for the risk of bilateral progression than in traditional nAMD-dominant models.

Regarding modifiable lifestyle factors, tobacco use demonstrated a significant association with bilateral conversion in the univariate analysis; however, it did not retain independent significance in the adjusted model. This attenuation may be attributed to our limited sample size and the retrospective nature of the study, where tobacco history might be underreported due to recall bias. Although the exact direction and magnitude of this bias remain uncertain, tobacco use remains a biologically plausible risk factor consistent with larger epidemiological cohorts [24,38]. Furthermore, our multivariable logistic regression (adjusted for sex and tobacco use) confirmed that baseline RPE abnormalities in the fellow eye are a significant independent risk factor for bilateral conversion (aOR 19.04, *p* = 0.032), a finding that is consistent with previous studies [39,40,41,42]. This finding highlights RPE integrity as a critical structural biomarker for progression, suggesting that localized anatomical changes may carry greater predictive weight than systemic factors in this mixed Asian cohort. In addition to these structural predictors, although prior studies have reported earlier detection and better baseline visual acuity in SEs, such differences were not observed in our cohort, possibly due to the small sample size [18,29,43,44].

Regarding clinical manifestations at the time of SE conversion, our data suggest two potential patterns of progression: an FE inactive group, characterized by a longer interval where the FE had already transitioned to quiescent fibrosis, and an FE active group, involving a shorter interval where the FE remained exudative, necessitating concurrent bilateral therapy (Table 3). Although not reaching statistical significance (*p* = 0.116), the FE active group showed a higher numerical frequency of annual outpatient visits between FE diagnosis and SE conversion compared to the inactive group (10.29 ± 5.02 vs. 4.40 ± 2.71). Collectively, we also observed a significant surge in annual cumulative IVI frequency, which escalated from 2.25 ± 2.55 injections during the unilateral phase to 7.75 ± 3.20 injections following SE involvement (*p* = 0.002). This 3.4-fold escalation in treatment burden underscores the substantial physiological and psychological toll on patients when both eyes require active therapy. Notably, 60% (3/5) of patients in the FE active group sought medical attention earlier than scheduled due to self-perceived visual changes in the SE. In contrast, while the annual cumulative number of IVIs in the FE inactive group is expectedly lower, this specific subgroup remains underrepresented in current literature. Whether the longer intervals or potential loss to follow-up in the FE inactive group contribute to delayed SE detection and reduced treatment outcomes warrants further investigation through larger-scale studies and longitudinal tracking.

However, as there were only eight bilateral cases, we explicitly acknowledge that these patterns and the observed 3.4-fold increase in cumulative treatment burden may be influenced by random variability rather than representing distinct biological entities. These findings should be interpreted as preliminary clinical observations that require validation in larger cohorts. Regarding logistical management, four of the five bilateral cases (80%) elected to switch from separate treatment schedules to same-day bilateral injections, whereas one patient (20%) maintained asynchronous treatment due to psychological concerns. No ocular or systemic adverse events were observed in eyes receiving same-day bilateral injections. These findings are consistent with prior reports demonstrating that same-day bilateral intravitreal anti-VEGF injections are generally safe when each eye is prepared and treated as a separate sterile procedure [45,46]. Ocular adverse events, including endophthalmitis, are rare, and no consistent reports of bilateral endophthalmitis have been described [45,46]. In addition, available evidence suggests a low incidence of systemic adverse events and all-cause mortality, although larger studies with longer follow-up are required to adequately assess rare systemic risks [47,48].

Beyond individual clinical factors, our findings reveal a potential ‘treatment gap’ in the era of next-generation anti-VEGF therapies. Although the majority of patients in this cohort were managed with ranibizumab or aflibercept 2 mg, newer agents such as aflibercept 8 mg or faricimab have demonstrated the potential to extend dosing intervals to every 16 weeks or even every 24 weeks in clinical trials [49,50]. While earlier studies noted that standard treat-and-extend regimens could compress the window for SE detection, we speculate that as these long-acting therapies become standard, the resulting decrease in clinic visit frequency might further delay the detection of SE conversion [17,18]. Notably, our data showed that SE conversion occurred within 2.0 years of the initial FE diagnosis, a timeframe that aligns with major Western cohorts [18,38,51]. Proactive monitoring is vital when therapeutic intervals are extended to reduce the FE treatment burden; such vigilant surveillance ensures prompt detection of bilateral disease despite reduced clinic visit frequency. This remains particularly critical for individuals with baseline SE RPE abnormalities, ensuring that the detection of SE conversion is not compromised by the use of long-acting therapies. Ultimately, future multicenter studies are warranted to confirm these associations and to optimize monitoring protocols for these evolving therapeutic regimens.

Despite the insights provided, several limitations warrant consideration when interpreting these findings. First, the retrospective design and relatively small sample size may limit statistical power; furthermore, as a single-center study, a degree of monocentric treatment bias may exist. A key methodological limitation is the differing observation durations between the two groups; while the timeline for the bilateral group was defined by the interval to conversion, the duration for the unilateral group represented total follow-up time. This discrepancy, though common in retrospective real-world research, may affect the direct comparability of risk profiles between cohorts. Additionally, all patients were managed under Taiwan’s NHI framework, which restricts initial agent access and prohibits switching anti-VEGF agents for the same eye during a treatment course. Spanning from the introduction of ranibizumab (2011) and aflibercept (2014) to faricimab (2024), this long-term study involved evolving reimbursement policies. Consequently, patients may have received different agents for each eye, including occasional off-label bevacizumab. This pharmacological heterogeneity likely explains the comparable visual acuity outcomes between the FE and SE, as eyes were not consistently treated with the same agents. Such diversity further limits the comparability of annual injection frequencies, which are inherently influenced by the distinct potencies and durability of the various therapies utilized over time. Finally, certain potentially relevant variables were not systematically available for analysis, including genetic susceptibility, detailed nutritional status, and viral infection histories [25,52,53,54,55]. Specifically, while all patients received standardized counseling for lutein supplementation at 10 milligrams per day, individual adherence could not be measured [54]. 

## 5. Conclusions

Our findings provide preliminary evidence of the heterogeneous patterns of bilateral nAMD and the subsequent surge in treatment burden. The significant 3.4-fold escalation in annual IVI frequency following SE conversion underscores the immense physiological and psychological toll on patients. Notably, our analysis encompassed the full spectrum of bilateral nAMD, including cases where the FE remained inactive during SE involvement.

Beyond the quantified burden, our study highlights a critical disparity in monitoring vigilance based on FE status. While 60% of patients in the FE active group sought medical attention earlier than their scheduled visits due to self-perceived SE visual changes—with 80% (4/5) opting for same-day bilateral injections to consolidate logistical demands—the lower monitoring frequency in the FE inactive group poses a distinct risk for delayed SE detection. These observations suggest that management strategies must evolve to account for FE activity status alongside the advent of long-acting anti-VEGF agents. Beyond drug efficacy, clinical protocols must integrate proactive patient education and adaptive surveillance to bridge the “vigilance gap” in varying bilateral treatment patterns. Ultimately, while this study offers a preliminary framework, larger prospective trials are warranted to further investigate these complex treatment behaviors in the era of advanced therapeutics.

## Figures and Tables

**Table 1:** Baseline demographic and clinical characteristics of patients with unilateral and bilateral neovascular age-related macular degeneration (nAMD).

Characteristic	Unilateral nAMD (*n* = 18) Mean ± SD/*n* (%)	Bilateral nAMD (*n* = 8) Mean ± SD/*n* (%)	*p* Value ****
Age (years)	72.69 ± 9.39	70.12 ± 5.99	0.49
Sex (Male)	8 (50.0)	7 (87.5)	0.09
Follow-up duration (years) *	2.91 ± 2.23	3.65 ± 3.16	0.51
*nAMD Subtype*			
Typical nAMD	11 (61.1)	6 (75.0)	0.81
PCV	7 (38.9)	2 (25.0)	
Primary IVI ** Agent			
Ranibizumab	11 (61.1)	5 (62.5)	0.96
Aflibercept	7 (38.9)	3 (37.5)	
*Systemic comorbidities*			
Diabetes mellitus	6 (33.3)	3 (37.5)	0.68
Hypertension	9 (50.0)	5 (62.5)	0.56
Dyslipidemia	4 (22.2)	5 (62.5)	0.09
Tobacco use	2 (11.1)	4 (50.0)	0.07
Baseline subfoveal second-eye RPE *** abnormality (OCT)	7 (38.9)	7 (87.5)	0.02

* Follow-up duration (years): For the unilateral group, defined as the interval from first-eye nAMD diagnosis to the last follow-up visit; for the bilateral group, defined as the interval from first-eye nAMD diagnosis to second-eye nAMD diagnosis. ** IVI: Intravitreal Injection. *** RPE = retinal pigmentary epithelium. **** Comparisons were performed using the chi-square test or Fisher’s exact test for categorical variables and Student’s *t*-test for continuous variables.

**Table 2:** Risk factors for progression to bilateral nAMD: univariate and multivariable logistic regression analysis (*n* = 26).

Variable	cOR *	95% CI ** for cOR	*p* Value	aOR ***	95% CI for aOR	*p* Value
Age	0.966	0.866–1.078	0.537			
Sex	0.114	0.012–1.131	0.064	0.105	0.006–1.737	0.115
DM	0.943	0.169–5.248	0.946			
HTN	1.667	0.303–9.157	0.557			
Dyslipidemia	4.333	0.742–25.294	0.103			
Tobacco	8.00	1.061–60.324	0.044	3.727	0.232–59.750	0.353
Initial SE RPE abnormality	11.00	1.103–109.674	0.041	19.038	1.287–281.535	0.032

* cOR = crude odds ratio; ** CI = confidence interval. Unilateral nAMD served as the reference group. *** aOR = adjusted odds ratio, adjusted for Sex, Tobacco and initial retinal pigmentary epithelial abnormality of the second eye.

**Table 3:** Clinical patterns of bilateral neovascular age-related macular degeneration and changes in intravitreal injection frequency.

Patient ID	Interval Between FE * and SE ** Diagnosis (Years)	FE Status at SE Diagnosis	Tobacco Use (Y/N)	Baseline Second-Eye RPE *** Abnormality at FE Diagnosis (Y/N)	Same-Day Bilateral Injections	Annual IVI **** Rate Before SE Diagnosis (FE Loading × 3 Y/N)	Annual IVI Rate After SE Diagnosis (SE Loading × 3 Y/N)
1	4.9	Subretinal scar	Y	Y	N	0 (Y)	7 (Y)
2	8	Subretinal scar	N	Y	N	0 (Y)	6 (Y)
3	5.7	Subretinal scar	N	Y	N	0 (Y)	4 (Y)
4	5.5	Active	Y	Y	Y	0 (N)	4 (N)
5	1.2	Active	Y	Y	Y	5 (Y)	12 (Y)
6 ^#^	0.7	Active	N	Y	N	4 (Y)	7 (Y)
7	1.8	Active	N	Y	Y	3 (N)	11 (Y)
8	0.7	Active	Y	N	Y	6 (Y)	11 (Y)

Notes. Y/N = yes/no; * FE = first eye; ** SE = second eye; *** RPE = retinal pigmentary epithelium; **** IVI = intravitreal injection. Annual IVI rates are expressed as injections per year at the patient level. ^#^ Case 6: SE treated with 4 faricimab injections after 2024; aflibercept used for all FE treatments.

## Data Availability

The datasets generated and/or analyzed during the current study are available from the corresponding author upon reasonable request.

## Abbreviations

The following abbreviations are used in this manuscript:

AMD	age related macular degeneration
nAMD	neovascular age related macular degeneration
Anti-VEGF	anti-vascular endothelial growth factor
BCVA	best corrected visual acuity
CFT	central foveal thickness
FE	first eye
IVI	intravitreal injection
NHI	national health insurance
OCT	optical coherence tomography
PCV	polypoidal choroidal vasculopathy
PRN	pro re nata
RPE	retinal pigment epithelium
SE	second eye
